# Supplementary material for: Structural and electronic features enabling delocalized charge-carriers in CuSbSe2
Source: Nat Commun. 2025 Jan 2;16:65. doi: 10.1038/s41467-024-55254-2 (PMC11697385; doi:10.1038/s41467-024-55254-2)
Supplement: Supplementary file 1 — Supplementary Information [file 41467_2024_55254_MOESM1_ESM.pdf]

**Supplementary Information for:**

# Structural and Electronic Features Enabling Delocalized Charge-Carriers in $CuSbSe_2$

Yuchen Fu,[1,†] Hugh Lohan,[1,2,†] Marcello Righetto,[3] Yi-Teng Huang,[1,4] Seán R. Kavanagh,[5] Chang-Woo Cho,[6,7] Szymon J. Zelewski,[4,8] Young Won Woo,[2,9] Harry Demetriou,[2,10] Martyn A. McLachlan,[2] Sandrine Heutz,[2,10] Benjamin A. Piot,[6] David O. Scanlon,[11] Akshay Rao,[4] Laura M. Herz,[3,12] Aron Walsh,[2] and Robert L. Z. Hoye[1,*]

1. Inorganic Chemistry Laboratory, University of Oxford, South Parks Road, Oxford OX1 3QR, United Kingdom
2. Department of Materials and Centre for Processable Electronics, Imperial College London, Exhibition Road, London SW7 2AZ, United Kingdom
3. Department of Physics, University of Oxford, Clarendon Laboratory, Parks Road, Oxford, OX1 3PU, United Kingdom
4. Cavendish Laboratory, University of Cambridge, JJ Thomson Ave, Cambridge CB3 0HE, United Kingdom
5. Harvard University Center for the Environment, Cambridge, Massachusetts 02138, United States

6. Laboratoire National des Champs Magnétiques Intenses, CNRS, LNCMI, Université Grenoble Alpes, Université Toulouse 3, INSA Toulouse, EMFL, F-38042 Grenoble, France
7. Department of Physics, Chungnam National University, Daejeon, 34134, Republic of Korea
8. Department of Experimental Physics, Faculty of Fundamental Problems of Technology, Wrocław University of Science and Technology, Wybrzeże Wyspiańskiego 27, 50-370 Wrocław, Poland
9. Department of Materials Science and Engineering, Yonsei University, Seoul 03722, Republic of Korea
10. London Centre for Nanotechnology, Imperial College London, Prince Consort Road, London, SW7 2AZ, United Kingdom
11. School of Chemistry, University of Birmingham, Birmingham, B15 2TT, United Kingdom
12. Institute for Advanced Study, Technical University of Munich, Lichtenbergstrasse 2a, D-85748 Garching, Germany

[†]These authors contributed equally to this work.
**Email:** robert.hoye@chem.ox.ac.uk (R.L.Z.H.)

**Supplementary Table 1 | Comparison of key properties of $CuSbSe_2$ with other Sb- and Bi-based compounds.** These are the charge-carrier mobility at room temperature, effective mass, bandgap and dielectric constants, along with the time taken for the photoconductivity signals to decay by 50% from the initial peak value ($t_{50}$) from OPTP measurements. The charge-carrier mobilities shown are extracted from the photoconductivity spectra measured by OPTP.

| Material | OPTP Mobility ($cm^2 \cdot V^{-1} \cdot s^{-1}$) | Effective mass ($m_h^*/m_0$; $m_e^*/m_0$) | Bandgap (eV) | Dielectric constant ($\epsilon_\infty$; $\epsilon_{stat}$) | $t_{50}$ (ps) |
|---|---|---|---|---|---|
| Polycrystalline $CuSbSe_2$ (this work) | 4.7±0.2 | 1.60; 0.43 | 0.9-1.2 | 11.3; 23.0 | 6.7 |
| Single crystal $CuSbSe_2$[1] | 87 (measured by Hall effect measurements) | | | | |
| $NaBiS_2$[2] | 0.29 (delocalized); 0.03 (localized) | 1.04; 0.24 | 1.4 | $\epsilon_\infty$ = 8.1; $\epsilon_{stat}$ = 43.7 | ~0.5 |
| $AgBiS_2$[3,4] | As-prepared: 0.43 ± 0.05 (delocalized), 0.11 ± 0.05 (localized); annealed: 2.70 ± 0.10 (delocalized), 2.20 ± 0.10 (localized) | 0.51; 0.24 | 1.45 | 19.43(*x*/*y*), 12.44(*z*); 115.61(*x*/*y*), 35.08(*z*) | <1 (as-prepared); 20-30 (heat treated) |
| $Cs_2AgSbBr_6$[5,6] | 0.5 (delocalized); 0.1 (localized) | 0.234–0.969; 0.289–0.431 | 1.64 | 4.82; 13.69 | 1–2 |
| $Cs_2AgBiBr_6$[7-11] | 3 (delocalized); 1.3 (localized) | 0.14; 0.37 | 2–2.25 | 4.60; 12.76 | 1–2 |
| Polycrystalline BiOI[12,13] | ~3 | 0.26; 0.23 | 1.93 | 8.60; 43.33 | 200–300 |
| Single crystal BiOI[12] | 26 (perpendicular), 83 (parallel), | | | | |

| | | | | | |
|---|---|---|---|---|---|
| | measured by time of flight measurements | | | | |
| $Sb_2S_3$[14-16] | 0.9±0.1 | 0.64; 0.40 | 1.7–1.8 | 11.55(*x*), 10.97(*y*), 8.25(*z*); 98.94(*x*), 94.21(*y*), 13.14(*z*) | 10–20 |
| $MA_3Bi_2I_9$[17-19] | Not reported | 0.95; 0.54 | 2.1–2.2 | 5.43(*x*), 4.67 (*z*); 39.89(*x*), 9.62(*z*) | Not reported |
| $Cs_3Bi_2I_9$[20,21] | Not reported | 0.94 (perpendicular to (100)), 2.14 (perpendicular to (001)); 0.33 (perpendicular to (100)), 3.22 (perpendicular to (001)) | 1.95 | 5.38 (perpendicular to (100)), 4.09 (perpendicular to (001)); 9.90 (perpendicular to (100)), 7.85 (perpendicular to (001)) | Not reported |
| BiSI[22-25] | Not reported | 0.95; 0.53 | 1.57 | 8.03; 37.81 | Not reported |
| $BiI_3$[26-29] | Not reported | 2.01; 0.68 | 1.8 | 7.1 (in plane), 6.4 (out of plane); 54 (in plane), 8.6 (out of plane) | Not reported |

## Supplementary Note 1 | Optimization of $CuSbSe_2$ thin film synthesis

Pawley fitting was performed to understand the phase-purity of the solution processed $CuSbSe_2$ thin film, which was thermally treated at 100 °C for 2 min in a glovebox then crystallized at 400 °C for 2 min in a tube furnace. The reference pattern used for $CuSbSe_2$ was obtained from the Inorganic Crystal Structure Database (ICSD),

collection code 418754. After excluding the two silicon substrate peaks, all other peaks measured were accounted for by the reference pattern, with mostly featureless residuals, and a goodness of fit (GOF) of 1.18. Together, these indicate the thin films to be phase-pure. Pawley fitting rather than Rietveld refinement was used for this analysis because of the high degree of preferred orientation in the thin films (Supplementary Table 2).

We also investigated the effect of heat treatment temperature on the phase purity of solution-processed $CuSbSe_2$ thin films. Initially, the spin-coated $CuSbSe_2$ films were thermally treated in a $N_2$-filled glovebox at 100 °C for 10 min. However, the XRD patterns had no clear, sharp peaks, indicating the films to be amorphous. We then investigated the spin-coated samples after heat treatment at 250 °C instead (also for 10 min), and obtained sharp XRD peaks, most of which matched with the reference pattern of $CuSbSe_2$. There were some minor peaks belonging to $Cu_3SbSe_3$ (ICSD database, coll. code 401095), as indicated by the blue dots in Supplementary Fig. 1c. To remove the $Cu_3SbSe_3$ skinnerite impurity phase, the sample was dried at 100 °C for 2 min in a $N_2$-filled glovebox, then heat treated at 400 °C for 2 min in an Ar-filled tube furnace. The XRD pattern of phase-pure sample is shown in Fig. 1b of the main text.

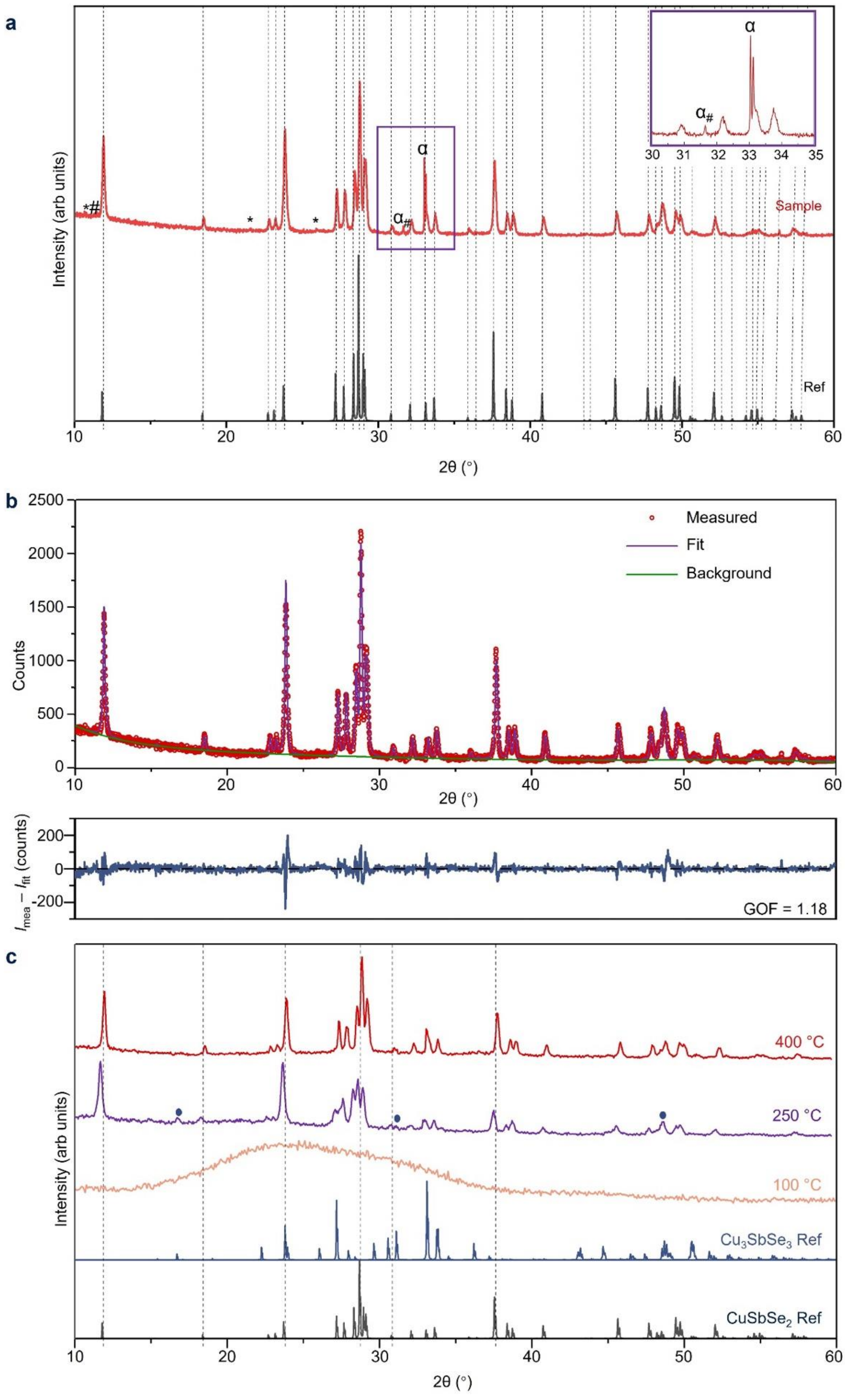


**Supplementary Fig. 1 | Analysis of X-ray diffraction (XRD) patterns of solution-processed $CuSbSe_2$ thin films. a,** XRD pattern (red, taken by the Bruker D8 Advance

system) of solution processed $CuSbSe_2$ thin film after heat treatment at 100 °C for 10 min then 400 °C for 2 min. Comparison with the $CuSbSe_2$ reference pattern (black, ICSD database, coll. code 418754). All $CuSbSe_2$ peaks are indicated by dashed lines. We also indicate the very minor peaks due to reflections from $K_\beta$ (*) and W $L_\alpha$ (#) radiation from the X-ray source, along with peaks from the crystalline silicon substrate (α) and the associated W $L_\alpha$ reflection ($\alpha_\#$). The inset shows a closeup of the peaks from the silicon substrate (α) and the associated W $L_\alpha$ reflection ($\alpha_\#$). **b,** Pawley fit of the XRD pattern shown in Supplementary Fig. 1a. The goodness of fit (GOF) is given, along with the residuals (blue), measured data points (red), fit (purple) and background (green). Please note that a profile fit was made to the measured pattern, and the silicon substrate peaks were removed in order to understand how well the reference pattern matches with the diffraction pattern from the thin film itself. Without removing these silicon substrate peaks, the GOF from Pawley fitting is 1.58, and the residuals are dominated by the silicon substrate peaks not fit. **c,** Comparison of the XRD patterns of films thermally treated at 400 °C for 2 min (red), 250 °C for 10 min (purple) and 100 °C for 10 min (pink) with the $CuSbSe_2$ reference pattern (black, ICSD database, coll. code 418754) and $Cu_3SbSe_3$ reference pattern (blue, ICSD database, coll. code 401095). The three XRD patterns were taken by the Bruker D2 Phaser system.

We also investigated the preferred orientation of the phase-pure $CuSbSe_2$ thin film prepared by thermal treatment at 400 °C by calculating the texture coefficients of the main peaks. The texture coefficient of crystal plane (*hkl*) ($\mathrm{TC}_{hkl}$) can be calculated using Eq. S1[30]:

$$\mathrm{TC}_{hkl} = \frac{\frac{I(hkl)}{I_0(hkl)}}{\frac{1}{N}\sum_{i=1}^{N}\frac{I(h_ik_il_i)}{I_0(h_ik_il_i)}} \qquad \text{(S1)}$$

where $I(h_ik_il_i)$ is the measured peak intensity of the $(h_ik_il_i)$ plane while $I_0(hkl)$ represents the standard peak intensity of the corresponding plane, and $N$ is the number of peaks considered. In this work, the standard peak intensity was calculated according

to the reference pattern from the ICSD database, with crystal planes (002), (004), (013) and (015) considered. As Supplementary Table 2 shows, the texture coefficients of planes (002) and (004) are higher than 1, indicating that the solution-processed $CuSbSe_2$ films have *c*-axis preferred orientation.

**Supplementary Table 2 |** Texture coefficients of crystal planes in solution-processed $CuSbSe_2$ thin film.

| Crystal plane | Texture coefficient |
|---|---|
| (002) | 1.93±0.03 |
| (004) | 1.30±0.07 |
| (013) | 0.37±0.02 |
| (015) | 0.40±0.08 |

**Supplementary Note 2 | Analysis on the phonon modes of $CuSbSe_2$**

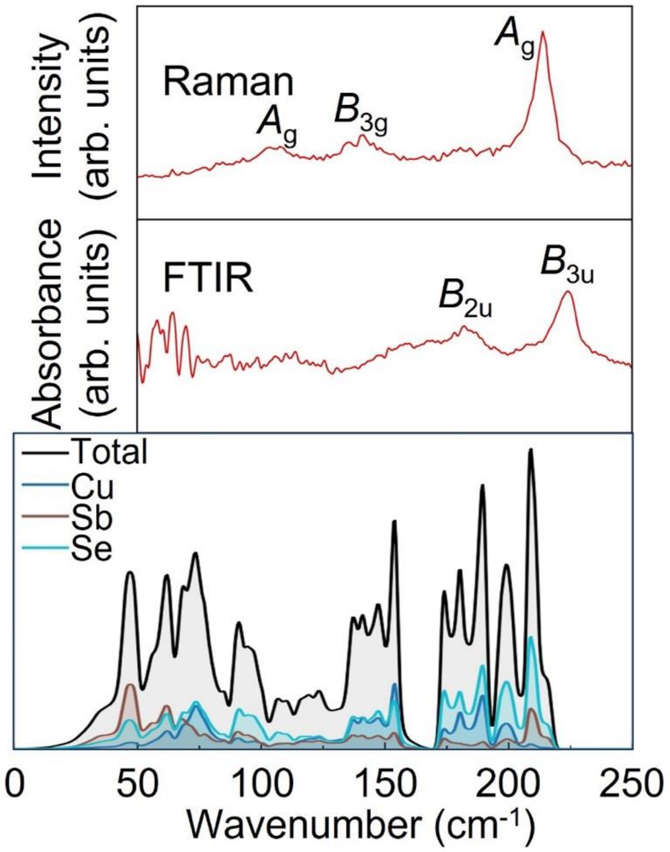


**Supplementary Fig. 2 |** Phonon density of states curves of $CuSbSe_2$ (bottom panel), along with the measured FTIR (middle panel) and Raman spectra (top panel) of a solution-processed $CuSbSe_2$ thin film. The phonon modes of intense peaks are labelled. The symmetries of the phonon modes were obtained by energy matching with the calculated phonon dispersion curve. The assignment of the $B_{2u}$ mode is tentative, since a $B_{3u}$ mode of similar energy is present. But the higher-energy $B_{3u}$ mode is assigned with greater confidence, since the nearest IR-active mode is 5 $cm^{-1}$ away.

**Supplementary Table 3 | Calculated symmetry operations and wavenumbers of all phonon modes of $CuSbSe_2$** excluding $B_{1u}$, $B_{2u}$, and $B_{3u}$ translational modes. Phonon modes observed in Raman and FTIR spectra are highlighted in bold.

| | Symmetry | Wavenumber ($cm^{-1}$) | | | |
|---|---|---|---|---|---|
| Raman active | $A_g$ | 49.5 | 63.9 | 88.3 | **104.4** |
| | | 152.0 | 185.6 | 209.2 | **212.9** |
| | $B_{1g}$ | 27.7 | 76.8 | 149.3 | 192.2 |
| | $B_{2g}$ | 52.4 | 68.5 | 103.2 | 125.5 |
| | | 145.1 | 194.4 | 202.1 | 211.7 |
| | $B_{3g}$ | 31.9 | 71.8 | **137.8** | 192.6 |
| IR active | $B_{1u}$ | 41.2 | 108.9 | 123.2 | 143.4 |
| | | 191.4 | 201.6 | 215.9 | |
| | $B_{2u}$ | 56.6 | 141.1 | **183.0** | |
| | $B_{3u}$ | 79.4 | 88.4 | 106.4 | 147.5 |
| | | 185.7 | 208.2 | **220.1** | |
| Inactive | $A_u$ | 26.4 | 70.8 | 139.6 | 183.7 |

**Supplementary Note 3 | Chemical and Structural Stability of $CuSbSe_2$**

*Chemical Stability*: To examine the chemical stability of the $CuSbSe_2$ thin films, we performed X-ray photoelectron spectroscopy (XPS) measurements on as-prepared and aged $CuSbSe_2$ samples. All samples were prepared at the same time with the optimized procedures described in Methods. Some samples were immediately measured by XPS (i.e., as prepared), while other samples were kept in ambient air (room temperature and approximately 80% relative humidity) for 3 weeks before XPS measurements (i.e., aged).

We obtained the survey spectra, Cu 2*p* core levels and Cu LMM Auger spectra, as well as the Sb 3*d* core levels (Supplementary Fig. 3–5). Supplementary Fig. 3 compares the XPS survey spectra of as-prepared and aged $CuSbSe_2$ thin films. After ageing, some peaks showed different intensities, but the peak positions and overall survey spectra had no significant changes.

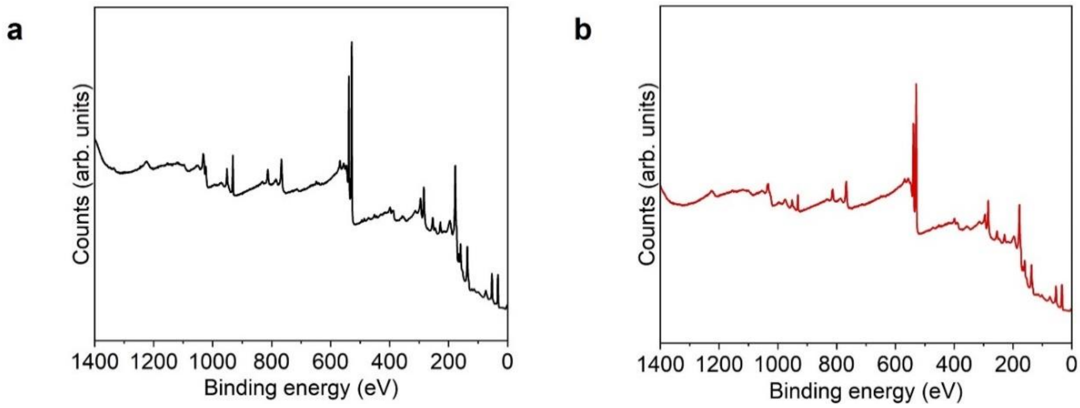


**Supplementary Fig. 3 | XPS survey spectra of $CuSbSe_2$ thin films.** Measurements for **a,** as-prepared and **b,** aged $CuSbSe_2$ samples.

To examine the oxidation states and bonding environments of the cations in $CuSbSe_2$ thin films more closely, we firstly analyzed the Cu 2*p* core levels and Cu LMM Auger spectra. As shown in Supplementary Fig. 4a and b, after three weeks of air exposure, the Cu 2*p* core levels and Cu LMM Auger spectra of the aged $CuSbSe_2$ thin films had no obvious changes compared to those of the as-prepared samples. To confirm the valence of the Cu species, the satellite found in the Cu 2*p* spectra is usually used, as indicated in Supplementary Fig. 4a[31-34]. The Cu 2*p* spectrum exhibits weak satellite signals, indicating that Cu remains in the +1 oxidation state after ageing. Also, the Cu LMM Auger spectra (Supplementary Fig. 4b) were similar to the spectra of other Cu(I) species in different compounds[35]. The kinetic energy of the peak was fitted to be 917.7±0.2 eV, close to the reported kinetic energy of Cu LMM peaks in $Cu_2Se$ (917.5 eV) and CuAgSe (917.6 eV)[36]. These results show that the Cu(I) species in this material are indeed stable, and remain in the same tetrahedral bonding environment in air over time.

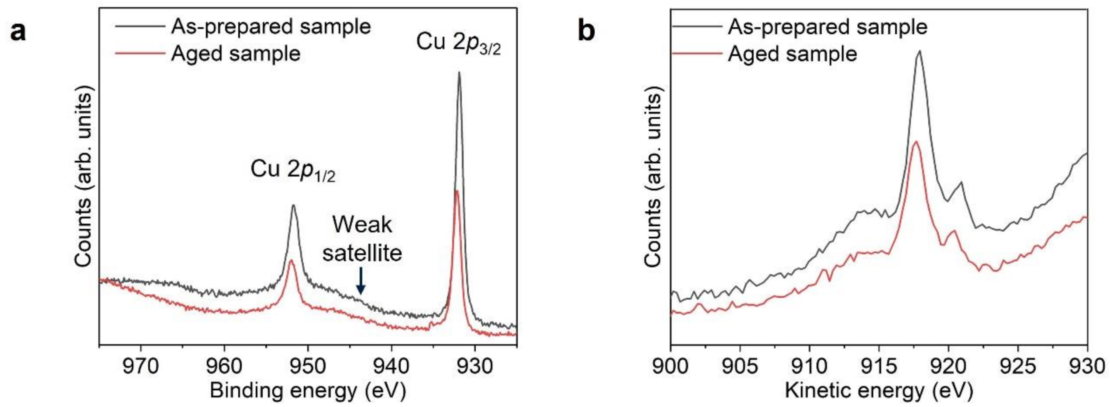


**Supplementary Fig. 4 | XPS spectra of $CuSbSe_2$ thin films (as-prepared and aged).** **a,** Cu 2*p* core levels and **b,** Cu LMM Auger spectra of as-prepared (black line) and aged $CuSbSe_2$ samples (red line).

The Sb 3d core level spectra are shown in Supplementary Fig. 5. Each spectrum is comprised of a doublet with binding energies 538.2 eV ($3d_{3/2}$, red line) and 528.8 eV ($3d_{5/2}$, red line). These binding energies, as well as the separation of 9.4 eV between them, are consistent with Sb(III)[37]. Similar Sb 3*d* doublets were also reported for $CuSbSe_2$ grown by close-space sublimation and hot injection[38,39].

We also found that the Sb 3*d* spectra for both the fresh and aged samples could be fit with Sb bonded to Se (main peak, red line) and O (purple line). After 3 weeks of storage in ambient air, the Sb–O peak increased in size and slightly shifted in binding energy, while the peak due to Sb bonded to Se remained at the same energy. An O 1s peak also appeared in the Sb 3*d* spectra for the aged samples, consistent with the formation of Sb–O species at the surface of the $CuSbSe_2$ film. The position of the Sb-O species in the Sb $3d_{3/2}$ peak has a binding energy of 539.5 eV, which is closer to the Sb $3d_{3/2}$ peak

reported for $Sb_2O_3$ (539.8 eV) than for $Sb_2O_5$ (540.4 eV)[40]. This confirms that the Sb in our films remained in the +3 oxidation state, and likely formed $Sb_2O_3$ on the film surface.

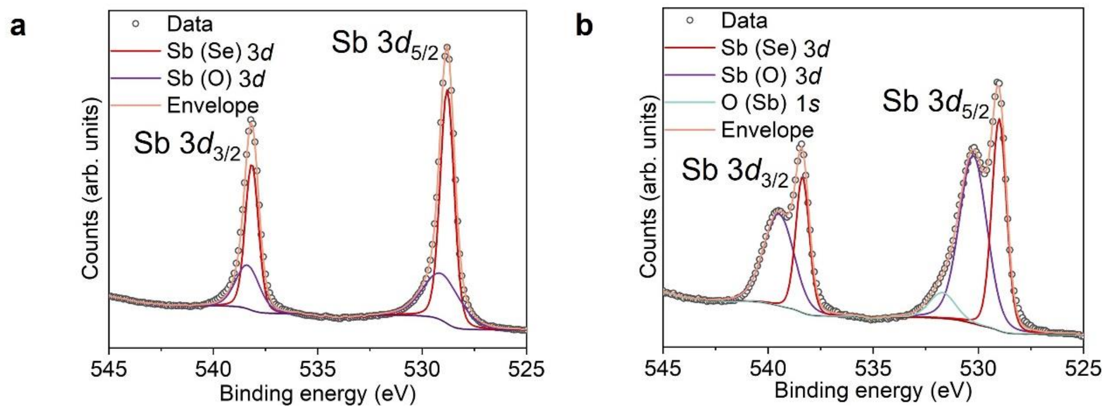


**Supplementary Fig. 5 | XPS spectra of $CuSbSe_2$ thin films, focussing on the Sb 3*d* peaks.** Measurements and fitting for **a,** as-prepared and **b,** aged $CuSbSe_2$ samples.

*XPS Methods* These XPS data were acquired using a Kratos Axis SUPRA using monochromated Al $K_\alpha$ (1486.69 eV) X-rays at 15 mA emission and 12 kV HT (180 W), and a spot size/analysis area of 700 × 300 μm$^2$. The instrument was calibrated to the Au 4*f* core level of a metal foil (83.95 eV), and dispersion adjusted give a binding energy of 932.6 eV for the Cu $2p_{3/2}$ line of metallic copper. The Ag $3d_{5/2}$ line FWHM at 10 eV pass energy was 0.544 eV. The source resolution for monochromatic Al $K_\alpha$ X-rays is ~0.3 eV. The instrument resolution was determined to be 0.29 eV at 10 eV pass energy using the Fermi edge of the valence band for metallic silver. The energy resolution with the charge compensation system on was <1.33 eV FWHM on PTFE. High resolution

core line spectra were obtained using a pass energy of 40 eV, step size of 0.1 eV and sweep time of 60 s, and for Auger spectra with a pass energy of 40 eV, step size of 0.25 eV and sweep time of 60s, resulting in a line width of 0.696 eV for Au $4f_{7/2}$. Survey spectra were obtained using a pass energy of 160 eV. The samples were electrically grounded to the instrument using copper clips. All data were recorded at a base pressure below $9 \times 10^{-9}$ Torr and temperature of 294 K. The data were analyzed using CasaXPS v2.3.19PR1.0. Peaks were fit with a Shirley background.

*Structural Stability:* We also examined the phase stability of the $CuSbSe_2$ thin films in diverse environments. For the stability test in an ambient environment, as-prepared and aged samples were prepared as described above for XPS measurements, and their XRD patterns were obtained. As shown in Supplementary Fig. 6a, after 3 weeks of storage in an ambient environment (room temperature and approximately 80% relative humidity), the XRD pattern of the $CuSbSe_2$ thin film was unchanged, showing the material to be phase-stable without encapsulation.

Another stability test was conducted at 85 °C and 85% relative humidity, and under 1-sun illumination. The details of the experimental methods can be found in our previous publication[41]. $CuSbSe_2$ thin films were kept under such conditions for 3, 8 and 24 hours,

respectively. The macroscopic appearance and XRD pattern of the $CuSbSe_2$ thin films were not changed after 24-hour exposure to this condition (Supplementary Fig. 6b). $CuSbSe_2$ therefore shows better stability under these conditions than triple-cation perovskite thin films, which began showing degradation products after only 8 h[41].

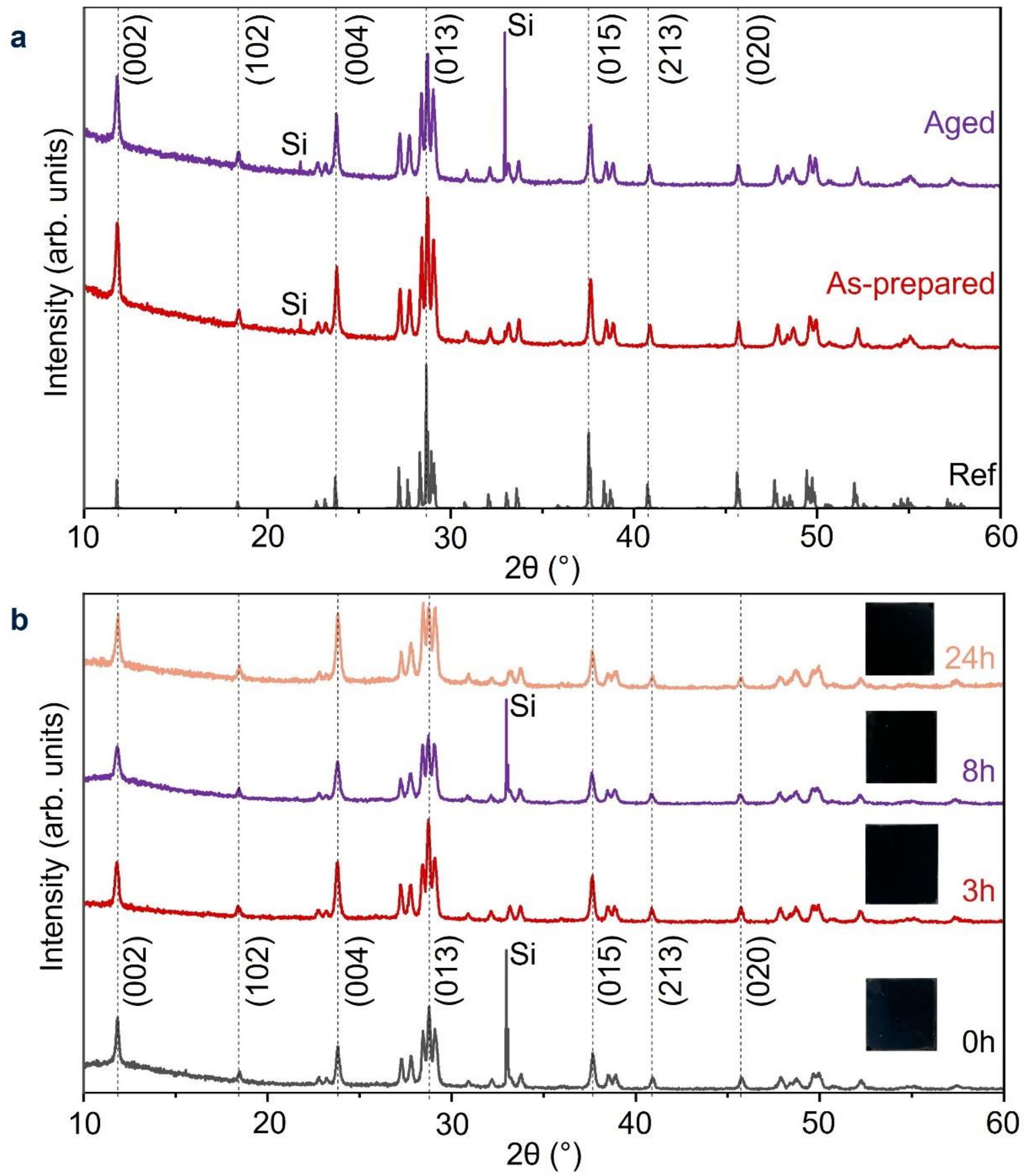


**Supplementary Fig. 6 | Phase stability of $CuSbSe_2$ thin films in diverse environments. a,** Comparison of the XRD patterns of as-prepared (red) and aged $CuSbSe_2$ films (stored at room temperature with approximately 80% relative humidity for 3 weeks, purple) with the $CuSbSe_2$ reference pattern (black, ICSD database, coll. code 418754). Main peaks are indicated by dashed lines along with the corresponding Miller indices. **b,** Appearance and XRD patterns of $CuSbSe_2$ thin films under 1-sun illumination, at 85 °C and 85% relative humidity over 24 h. Main peaks are indicated by dashed lines along with the corresponding Miller indices. The XRD peak from the Si substrate is indicated.

## Supplementary Note 4 | Optical absorption spectrum analysis

Supplementary Fig. 7a,b show the experimentally-determined optical absorption coefficient spectra of $CuSbSe_2$. The data in Supplementary Fig. 7a was obtained only by UV-visible (UV-vis) spectrophotometry measurements over a wide range of photon energies, while Supplementary Fig. 7b was obtained by combining photothermal deflection spectroscopy (PDS) measurements for photon energies below 1.3 eV, and UV-vis measurements for photon energies above 1.3 eV. UV-vis allows us to determine the absolute absorption coefficients, while the relative absorbance measured by PDS was scaled to match the absorption coefficient values determined by UV-vis at 1.3 eV. Supplementary Fig. 7c shows the calculated optical absorption coefficient spectrum. As discussed in the main text, both spectra exhibit a similar shoulder near the absorption onset, which is consistent with the shoulder originating from the electronic structure of $CuSbSe_2$.

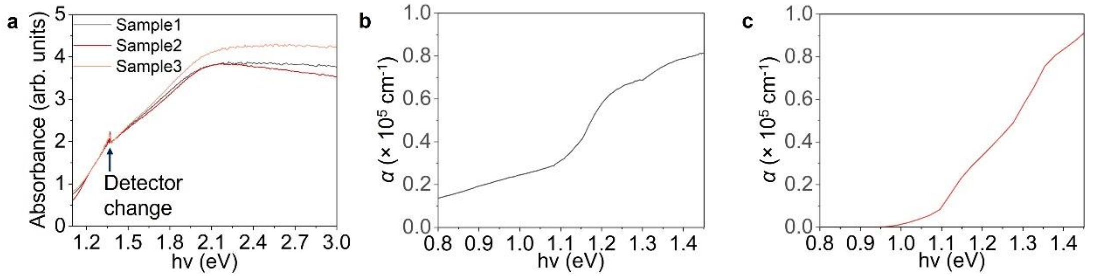


**Supplementary Fig. 7 | Optical absorption properties of $CuSbSe_2$. a,** Absorbance of three $CuSbSe_2$ thin film samples over a broad photon energy range. The three samples were prepared with identical parameters, and heat treated at 400 °C following our optimized procedure, as described in the main text. The peak at 1.37 eV (900 nm wavelength) was caused by a change in the detector in the instrument. **b,**

Experimentally-determined optical absorption coefficient spectrum. **c,** Calculated optical absorption coefficient spectrum.

Finally, we note that it is important that free charge-carriers rather than excitons form in $CuSbSe_2$ in order to use OPTP measurements to determine whether carrier localization occurs or not. This is because exciton formation would have also resulted in a rapid decay in the OPTP signal[42]. But in the case of $CuSbSe_2$, the exciton binding energy was low ($E_b$ <10 meV; refer to discussion around Fig. 1 in the main text). Furthermore, the fluence-independence of the OPTP signal decays (Fig. 3a) rules out significant contributions from exciton formation processes to the photoconductivity decay. Thus, if a rapid decrease in photoconductivity occurred, this could be unambiguously assigned to carrier localization.

*Optical Absorption Measurement Methods* Ultraviolet-visible spectrophotometry (UV-vis) absorption spectra were measured in air at room temperature using a Shimadzu UV 3600 spectrometer equipped with an integrating sphere (Supplementary Fig. 7a). Before the UV-vis measurements, the spectrometer was calibrated by setting the transmittance of the blank as 100% and the reflectance of the equipped $BaSO_4$ plate as 100%. The $CuSbSe_2$ film was deposited onto 1.2 × 1.2 $cm^2$ glass substrates. PDS measurements were taken at room temperature on a home-made setup detailed elsewhere[43]. $CuSbSe_2$

films were spin coated on 2 mm thick circular quartz substrates with 1 cm diameter (Spectrosil® 2000). The parameters of spin coating and heat treatment are described in the Supplementary Note 1. The samples were vacuum sealed in a $N_2$-filled glovebox before shipment between laboratories. When received, the sealed bag was opened in a $N_2$-filled glovebox and the samples were loaded into the sample holder chamber, then immersed in an inert liquid FC-72 Fluorinert™ (3M Company), which has a high thermo-optical coefficient. A monochromatic beam from a 250 W quartz tungsten halogen lamp (Newport-Oriel) integrated with a 250 mm focal length monochromator (CVI DK240) was illuminated perpendicularly to the sample surface, modulated with a mechanical chopper at a frequency of 13 Hz. Non-radiative recombination processes at the film surface led to an alternating temperature gradient, and thus a refractive index gradient in the liquid surrounding the sample in close proximity to the surface. A 670 nm CW diode laser beam passing through the immersive medium, parallel to the sample surface (the transverse configuration), changes its optical path synchronously with the modulating pump excitation. The probe beam deflection is detected by a quadrant photodiode, with the signal amplitude demodulated with a lock-in amplifier (Stanford Research Systems SR830). The relative absorbance spectrum measured by PDS was normalised to its highest signal value. We then mapped this maximum value of the absorbance, at 1.4 eV photon energy, onto the absolute absorption coefficient value

obtained from UV-vis at the same photon energy. The whole absolute absorption coefficient spectrum could then be acquired (Supplementary Fig. 7b). The details about determining the optical absorption coefficient by UV-vis are described in the Methods section of the main text.

**Supplementary Note 5 | Mobility Analysis**

***Extraction of charge-carrier mobility from OPTP measurements***

We extracted effective charge-carrier mobility following the procedure developed by Wright et al. and Buizza et al.[44,45]. The method is based on the approach developed by Wehrenfennig et al.[46] to estimate charge-carrier mobilities in semiconductor thin films. Briefly, for materials with thicknesses smaller than the wavelength of the incident THz radiation, we can retrieve the sheet photoconductivity from the fractional change in the transmitted THz electric field $\Delta T/T$, and we can express it as

$$\Delta S = -\epsilon_0 c(n_1 + n_3)\left(\frac{\Delta T}{T}\right) \tag{S2}$$

where $n_3$ = 2.13 and $n_1$ = 1 are the refractive indexes of quartz and vacuum, respectively[47].

Here, in order to derive the effective charge-carrier mobility from the sheet photoconductivity, we define the number of initially photogenerated charge-carriers as

$$N = \phi \frac{E\lambda}{\mathrm{h}c}\left(1 - R_{\mathrm{pump}} - T_{\mathrm{pump}}\right) \tag{S3}$$

where $\phi$ is the photon-to-charge branching ratio (i.e., the fraction of generated charges per absorbed photon absorbed), $E$ is the pump pulse energy, $\lambda$ is the excitation wavelength, and $R_{\mathrm{pump}}$ and $T_{\mathrm{pump}}$ are the reflectance and transmittance of the sample at the excitation wavelength (400 nm, 3.1 eV). Here, Eq. S2 and Eq. S3 can be used to extract the charge-carrier mobility $\mu$ through the relation

$$\mu = \frac{\Delta S A_{\text{eff}}}{Ne}, \qquad \text{(S4)}$$

where $A_{\text{eff}}$ is the effective overlap area between the THz and pump beam, and $e$ is the elementary charge. By substituting Eq. S2 and Eq. S3 into Eq. S4, we can obtain the effective (i.e., multiplied by the photon-to-charge branching ratio) charge-carrier mobility as:

$$\phi\mu = -\epsilon_0 \mathrm{c}(n_{\mathrm{q}} + n_{\mathrm{v}}) \frac{A_{\mathrm{eff}}\mathrm{hc}}{eE\lambda(1-R_{\mathrm{pump}}-T_{\mathrm{pump}})} \left(\frac{\Delta T}{T}\right). \qquad \text{(S5)}$$

We note that the sheet photoconductivity signal measured by OPTP arises from the contributions of both photogenerated free electrons and holes. Therefore, the extracted charge-carrier mobility is the effective electron-hole sum mobility.

***Temperature-dependent resistivity***

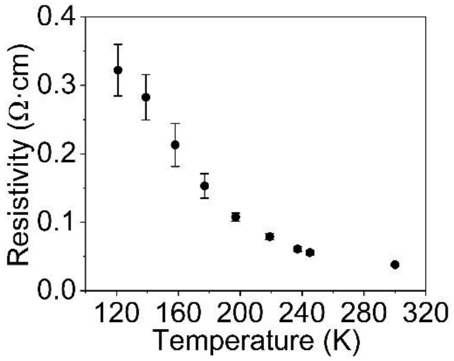


**Supplementary Fig. 8 | Temperature-dependent resistivity of $CuSbSe_2$** thin films determined using Hall effect measurements. The error bars represent the standard deviation between two samples, which were prepared and measured with identical parameters.

## Supplementary Note 6 | Long-time TA measurements

Long-time TA measurements are shown in Supplementary Fig. 9a-c. For pump-probe delays longer than 1 ns, the GSB signal could not be observed due to the strong PIA signal, which made it difficult to investigate the charge-carrier kinetics based on TA results.

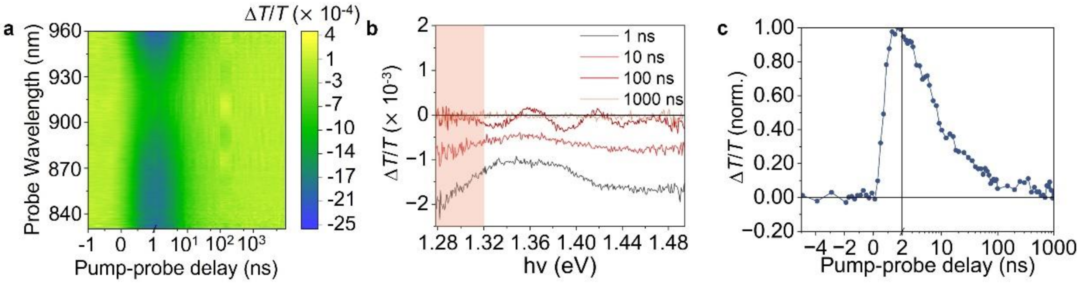


**Supplementary Fig. 9 | Long-time TA measurement results. a,** Long-time TA signal colour map of $CuSbSe_2$ films excited by 355 nm pump (800 ps pulse width, 21 μJ $cm^{-2}$ $pulse^{-1}$ fluence, 500 Hz repetition rate), along with **b,** TA spectra for different pump-probe delays from 1 to 1000 ns, and **c,** its normalized PIA signal kinetics. Please note that the signal in part **c** is normalized and is therefore not shown as negative values, as would conventionally be the case. The PIA kinetics were acquired by averaging the signals from 1.28 to 1.32 eV (pink shaded area in **b**) and normalized to the maximum $\Delta T/T$ value.

## Supplementary Note 7 | Computational analysis of relaxed deformation potentials in $CuSbSe_2$

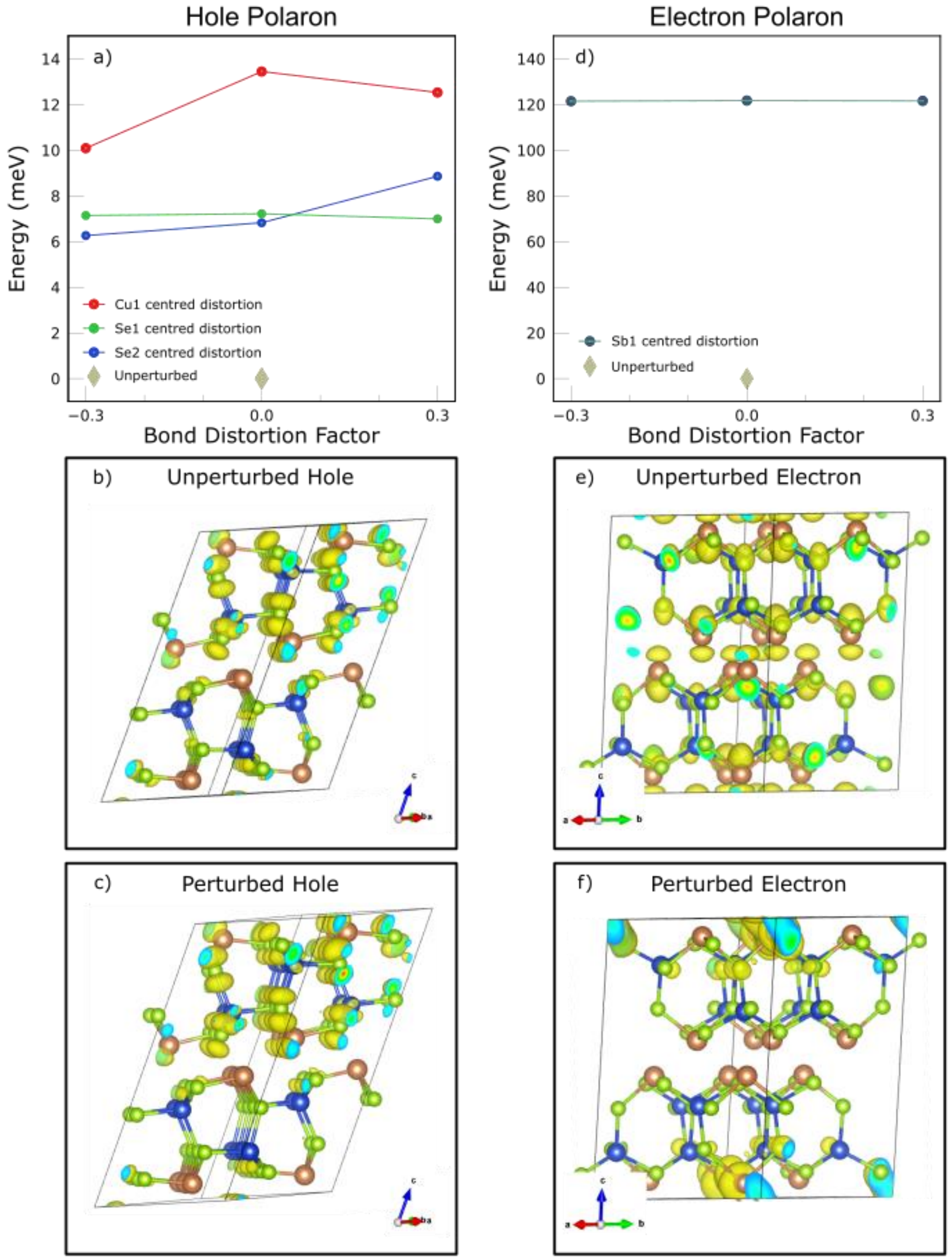


**Supplementary Fig. 10 | Computations to directly determine whether small polarons form in $CuSbSe_2$. a,** Addition of an unpaired hole followed by distortion around the Cu, Se1 and Se2 sites and/or rattling of the lattice results in a relatively small change in DFT total energy of about ~12 meV compared to the unperturbed state. Cu

and the two Se sites were selected, since the valence band is dominated by these states (refer to Fig. 2b in the main text) and may be oxidized. The partial electron density function of the unpaired hole for the **b,** unperturbed and the **c,** perturbed structures show a quasi-2D valence band as the electron density is confined to a single layer. **d,** Addition of an unpaired electron followed by distortion around the Sb site results in the formation of a metastable state 0.12 eV above the unperturbed structure. Analysis was performed on distortions around Sb because Sb 5*p* orbitals dominate the lower conduction band (refer to Fig. 2b in the main text) and Sb may be reduced. The partial electron density of the unpaired electron in the **e,** unperturbed structure shows a delocalized 3D solution, while the higher energy **f,** perturbed structure shows quasi-1D character because the electron density is confined to ribbons running through the interlayer space. In this analysis, we considered a supercell that was four times the volume of a unit cell, and we did not observe any localization of either the electron or hole charge density (0D character) to within one unit cell. That is, these computations support the conclusion that small polarons do not form in $CuSbSe_2$

The details of how these calculations were performed are included in the Methods

section of the main text.

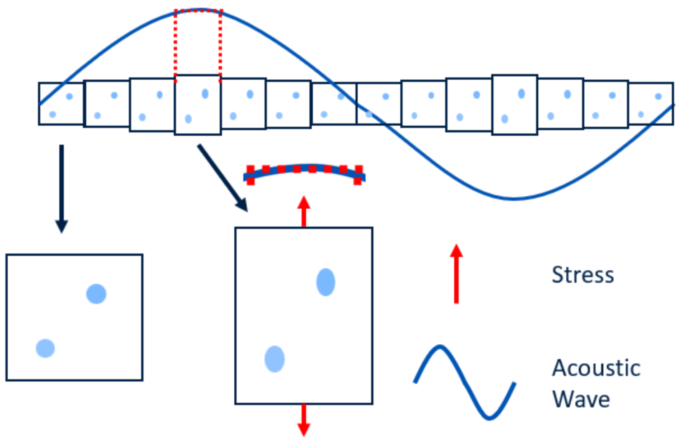


**Supplementary Fig. 11 | Illustrating the effects of acoustic waves in materials.** A long wavelength acoustic phonon propagating through a material can be approximated locally as a homogenous strain to the unit cell. This is one of the key assumptions made in deformation potential theory[48].

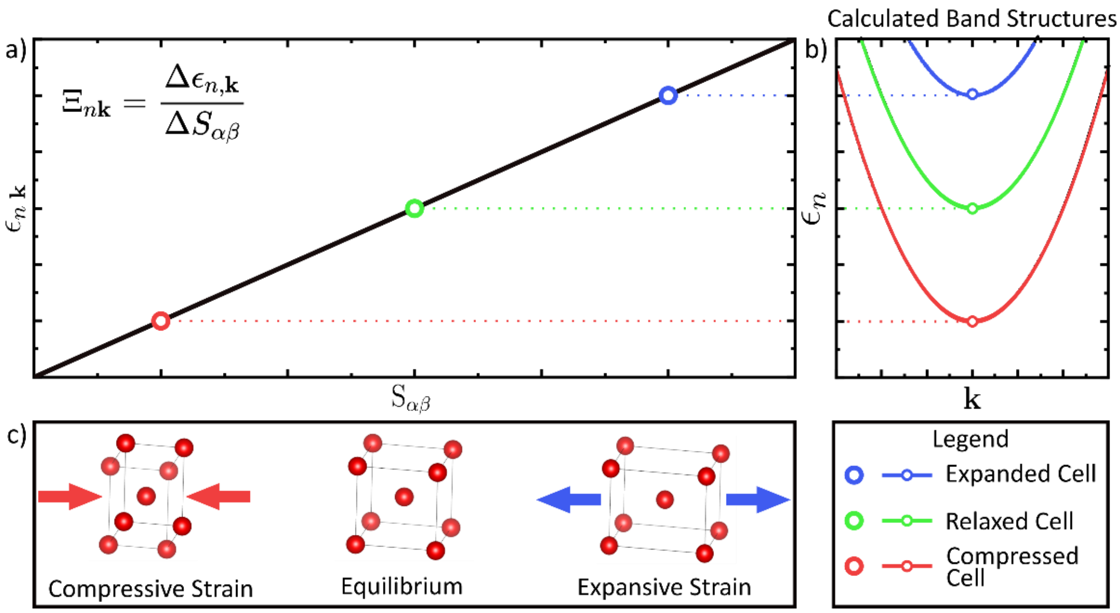


**Supplementary Fig. 12 | Schematic illustrating how the acoustic deformation potential of band *n* at point k was calculated using a modified method of Wei and Zunger[49]. a,** Plot of band energy $\epsilon_{n,\mathbf{k}}$ against homogenous strain $S_{\alpha\beta}$. The acoustic deformation potential is defined as the change in $\epsilon_{n,\mathbf{k}}$ with respect to $S_{\alpha\beta}$ **b,** Change in band edge energy of a simple parabolic band under axial compression (red) and expansion (blue). **c,** Uniaxial compression (red) and expansion (blue) of a BCC unit cell. Note, it is implicitly assumed in this schematic that the change in energy of core levels is negligible, and that the band edges are in equivalent electrostatic reference frames

Deformation potentials can be measured but are more readily calculated via ab-initio methods. The method of Wei and Zunger is explained in Supplementary Fig. 12. In this method, a fixed internal structure is maintained, which means in practice that the change in relative atomic coordinates of each species is not allowed in response to the deformation of the unit cell. That is, an unequilibrated structure is compared to the ground-state undeformed structure. Here we define an unequilibrated structure as one to which a homogenous strain has been applied, but the internal atomic coordinates have not subsequently been allowed to relax to a local minimum.

The use of unequilibrated structures was reasonable in the types of materials originally investigated by Wei and Zunger, which were high-symmetry zinc blende semiconductors with no internal structural degrees of freedom. However, this assumption may not be valid in the case of $CuSbSe_2$. We therefore performed additional deformation potential calculations at the $r^2$SCAN level. In the case of $CuSbSe_2$, we found that allowing atomic relaxations following the deformation of the unit cell results in a mean reduction in the predicted deformation potentials by –25 % and –12.4% for CBM and VBM, respectively, as compared to the unequilibrated case (Supplementary Table 4). To probe the cause of this reduction, we compared cation-anion bond lengths before and after atomic relaxation. We found that when the unit cell was strained along the *c*-axis and allowed to relax, there were comparatively little changes in the intralayer bond lengths because these distortions were mostly taken up by an increase in interlayer spacing (Fig. 4b). Referring to Figs. 2b and 4c in the main text, we see that the band edges are dominated by intralayer bonding, and so relaxing these bonds towards their ground state configurations should minimize changes in the electronic structure, thus minimizing the deformation potentials.

**Supplementary Table 4 | Complete list of deformation potential calculations.** Two groups of deformation potential calculations were carried out, those using the r$^2$SCAN functional, and those using the HSE06 functional. $i$ and $j$ refer to components of the symmetric $3 \times 3$ deformation potential tensor. Those carried out at r$^2$SCAN level were done so with both unequilibrated ($E_{\mathrm{d}}^{\prime\mathrm{unequil}}$), and equilibrated ($E_{\mathrm{d}}^{\mathrm{equil}}$) structure sets. This allowed calculation of relative change in deformation potentials after equilibration ($\Delta E_{\mathrm{d}} = 100\%.\frac{E_{\mathrm{d}}^{\prime\mathrm{equil}} - E_{\mathrm{d}}^{\prime\mathrm{unequil}}}{E_{\mathrm{d}}^{\prime\mathrm{unequil}}}$). From these results, we conclude that equilibration results in a mean reduction of deformation potentials for $CuSbSe_2$. For calculations at HSE06 level, deformation potentials are only calculated in the unequilibrated case as the expected mean reduction in would not affect our conclusions, but would add significant computational cost. The mean of each tensor ($E_{\mathrm{d}}^{\prime\mathrm{unequil}}, E_{\mathrm{d}}^{\prime\mathrm{unequil}}, \Delta E_{\mathrm{d}}$) is calculated as an average of its eigenvalues (refer to Supplementary Note 8).

| | **r$^2$SCAN** | | | | | | **HSE06** | |
|---|---|---|---|---|---|---|---|---|
| $ij$ | $E_{\mathrm{d,ij}}^{\mathrm{VBM,unequil}}$ (eV) | $E_{\mathrm{d,ij}}^{\mathrm{VBM,equil}}$ (eV) | $\Delta E_{\mathrm{d,ij}}^{\mathrm{VBM}}$ (%) | $E_{\mathrm{d,ij}}^{\mathrm{CBM,unequi}}$ (eV) | $E_{\mathrm{d,ij}}^{\mathrm{CBM,equil}}$ (eV) | $\Delta E_{\mathrm{d,ij}}^{\mathrm{CBM}}$ (%) | $E_{\mathrm{d,ij}}^{\mathrm{VBM,unequil}}$ (eV) | $E_{\mathrm{d,ij}}^{\mathrm{CBM,unequi}}$ (eV) |
| 11 | 0.46 | 0.35 | −23.9 | 0.2 | 0.21 | 5.0 | 1.16 | 6.60 |
| 22 | 0.75 | 0.58 | −22.7 | 6.5 | 7.6 | 16.9 | 1.93 | 6.32 |
| 33 | 1.87 | 1.34 | −28.3 | 2.05 | 0.84 | −59.0 | 2.11 | 6.62 |
| 12 | 0.65 | 0.09 | −86.2 | 0.21 | 0.13 | −38.1 | 1.77 | 6.45 |
| 13 | 3.17 | 3.6 | 13.6 | 1.21 | 0.98 | −19.0 | 4.66 | 4.58 |
| 23 | 0.02 | 0 | -100.0 | 0.8 | 0.14 | −82.5 | 1.22 | 6.3 |
| mean | 1.03 | 0.76 | −25.0 | 2.92 | 2.88 | −12.4 | 1.73 | 6.51 |

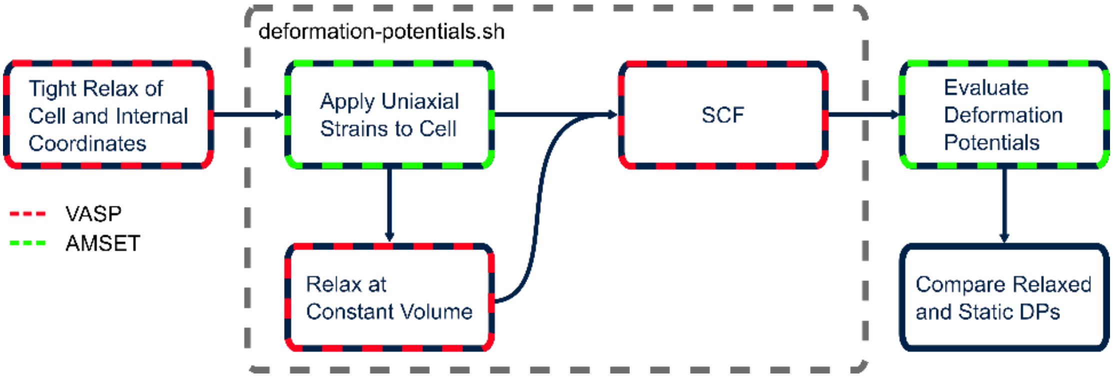


**Supplementary Fig. 13 | Comparison of equilibrated and unequilibrated deformation potentials workflow.** Computational workflow for the analysis of deformation potentials at $r^2$SCAN level. Deformation potentials were carried out with fixed internal structures (i.e., where the relative coordinate of each ion is not allowed to change upon distortion of the unit cell), and again where the ions were allowed to relax to their equilibrium positions. Calculations were semi-automated using AMSET, and the 'deformation-potential.sh' script available is among the raw data in the folder for Supplementary Table 4.

**Supplementary Table 5 | Born effective charge (BEC) of pnictogen atoms in Sb- and Bi-based compounds, along with the BEC value of Pb atom in $CH_3NH_3PbI_3$.** The label *a*, *b* and *c* refer to principal crystallographic axes, while *xx*, *yy* and *zz* refer to diagonal components of the Born effective charge tensors.

| Material | Born effective charge |
| --- | --- |
| $CuSbSe_2$ (this work) | Sb: 1.03 (*a*), 5.65 (*b*), 1.44 (*c*) |
| $Cs_2AgBiBr_6$[50] | Bi: 4.79 (average) |
| BiOI[51] | Bi: 5.87 (*a*); 5.87 (*b*); 3.08 (*c*) |
| $Sb_2S_3$[52] | Sb1: 2.89 (*xx*); 5.62 (*yy*); 7.36 (*zz*);<br>Sb2: 3.33 (*xx*); 7.25 (*yy*); 4.50 (*zz*) |
| $Cs_3Bi_2I_9$[53] | Bi: 3.9 (*xx*), 3.9 (*zz*) |
| BiSI[54] | Bi: 6.42 (*a*); 3.04 (*b*); 4.01 (*c*) |
| $BiI_3$[29] | Bi: 5.2 (*a*); 5.2 (*b*), 2.8 (*c*) |
| $CH_3NH_3PbI_3$[55] | Pb: 5.22 (*a*), 3.54 (*b*), 4.86 (*c*) |

## Supplementary Note 8 | Tensor averaging

Finding the average value for some quantities in Table 1 requires combining tensors of different ranks into a single value, e.g., deformation potentials ($E_{n\mathbf{k}}$) and elastic constants ($c_{\mathrm{ijkl}}$) are rank 2 and rank 3 tensors respectively. To arrive at the average shown in Table 1, a tensor averaging scheme was employed for each tensor, and these values were used in the relevant formula. For rank 2 tensors, the tensor was diagonalized and the average of their eigenvalues taken. There are a number of common schemes used to average the tensor of elastic constants (e.g., Reuss, Hill, and Voigt averages). For this work, all schemes produced similar values. We used the Reuss average, calculated via the Elate web app[56]. Simple means were used for rank 1 tensors, except for the effective masses, for which the harmonic mean was taken. Once a tensor

average for each quantity in each formula was found, these were used in the relevant equations to find the average values quoted in Table 1. A Jupyter notebook is available in the raw data file (in the Table 1 folder) which explicitly shows all data processing steps.

## Supplementary References


1 Chen, T. *et al.* Ultralow Thermal Conductivity and Enhanced Figure of Merit for $CuSbSe_2$ via Cd-Doping. *ACS Appl. Energy Mater.* **4**, 1637–1643 (2021). https://doi.org/10.1021/acsaem.0c02820

2 Huang, Y. T. *et al.* Strong absorption and ultrafast localisation in $NaBiS_2$ nanocrystals with slow charge-carrier recombination. *Nat. Commun.* **13**, 4960 (2022). https://doi.org/10.1038/s41467-022-32669-3

3 Righetto, M. *et al.* Cation-Disorder Engineering Promotes Efficient Charge-Carrier Transport in $AgBiS_2$ Nanocrystal Films. *Adv. Mater*. **35**, e2305009 (2023). https://doi.org/10.1002/adma.202305009

4 Liu, X., Xiao, H., Zang, Z. & Li, R. Atomic periodic engineering enabled ultrathin high-efficiency $AgBiS_2$ solar cells. *Chem. Commun.* **58**, 12066–12069 (2022). https://doi.org/10.1039/D2CC04610E

5 Wei, F. *et al.* Enhanced visible light absorption for lead-free double perovskite $Cs_2AgSbBr_6$. *Chem. Commun.* **55**, 3721–3724 (2019). https://doi.org/10.1039/C9CC01134J

6 Righetto, M. *et al.* Alloying Effects on Charge-Carrier Transport in Silver–Bismuth Double Perovskites. *J. Phys. Chem. Lett.* **14**, 10340–10347 (2023). https://doi.org/10.1021/acs.jpclett.3c02750

7 Bartesaghi, D. *et al.* Charge Carrier Dynamics in $Cs_2AgBiBr_6$ Double Perovskite. *J. Phys. Chem. C* **122**, 4809–4816 (2018). https://doi.org/10.1021/acs.jpcc.8b00572

8 Li, Z. *et al.* Bandgap lowering in mixed alloys of $Cs_2Ag(Sb_xBi_{1-x})Br_6$ double perovskite thin films. *J. Mater. Chem. A* **8**, 21780–21788 (2020). https://doi.org/10.1039/D0TA07145E

9 Pan, W. *et al.* $Cs_2AgBiBr_6$ single-crystal X-ray detectors with a low detection limit. *Nat. Photon.* **11**, 726–732 (2017). https://doi.org/10.1038/s41566-017-

0012-4
10 Bellakhdar, T., Nabi, Z., Bouabdallah, B., Benichou, B. & Saci, H. Ab-initio study of structural, electronic, mechanical and optical properties of the tetragonal $Cs_2AgBiBr_6$ halide double perovskite. *Applied Physics A* **128**, 155 (2022). https://doi.org/10.1007/s00339-022-05276-8
11 Wu, B. *et al.* Strong self-trapping by deformation potential limits photovoltaic performance in bismuth double perovskite. *Sci. Adv.* **7**, eabd3160 (2021). https://doi.org/10.1126/sciadv.abd3160
12 Jagt, R. A. *et al.* Layered BiOI single crystals capable of detecting low dose rates of X-rays. *Nat. Commun.* **14**, 2452 (2023). https://doi.org/10.1038/s41467-023-38008-4
13 Lal, S. *et al.* Bandlike Transport and Charge-Carrier Dynamics in BiOI Films. *J. Phys. Chem. Lett.* **14**, 6620–6629 (2023). https://doi.org/10.1021/acs.jpclett.3c01520
14 Wang, X., Li, Z., Kavanagh, S. R., Ganose, A. M. & Walsh, A. Lone pair driven anisotropy in antimony chalcogenide semiconductors. *Phys. Chem. Chem. Phys.* **24**, 7195-7202 (2022). https://doi.org/10.1039/d1cp05373f
15 Wang, X., Ganose, A. M., Kavanagh, S. R. & Walsh, A. Band versus Polaron: Charge Transport in Antimony Chalcogenides. *ACS Energy Lett.* **7**, 2954-2960 (2022). https://doi.org/10.1021/acsenergylett.2c01464
16 Jia, Z. *et al.* Charge-Carrier Dynamics of Solution-Processed Antimony- and Bismuth-Based Chalcogenide Thin Films. *ACS Energy Lett.* **8**, 1485-1492 (2023). https://doi.org/10.1021/acsenergylett.3c00140
17 Park, B.-W. *et al.* Bismuth Based Hybrid Perovskites $A_3Bi_2I_9$ (A: Methylammonium or Cesium) for Solar Cell Application. *Adv. Mater.* **27**, 6806-6813 (2015). https://doi.org/https://doi.org/10.1002/adma.201501978
18 Pazoki, M. *et al.* Bismuth Iodide Perovskite Materials for Solar Cell Applications: Electronic Structure, Optical Transitions, and Directional Charge Transport. *J. Phys. Chem. C* **120**, 29039–29046 (2016). https://doi.org/10.1021/acs.jpcc.6b11745
19 Zheng, X. *et al.* Ultrasensitive and stable X-ray detection using zero-dimensional lead-free perovskites. *Journal of Energy Chemistry* **49**, 299-306 (2020). https://doi.org/https://doi.org/10.1016/j.jechem.2020.02.049
20 Sun, Q. *et al.* Optical and electronic anisotropies in perovskitoid crystals of $Cs_3Bi_2I_9$ studies of nuclear radiation detection. *J. Mater. Chem. A* **6**, 23388–23395 (2018). https://doi.org/10.1039/C8TA09525F
21 Sun, Q. *et al.* Anisotropic dielectric behavior of layered perovskite-like $Cs_3Bi_2I_9$ crystals in the terahertz region. *Phys. Chem. Chem. Phys.* **22**, 24555–24560

(2020). https://doi.org/10.1039/D0CP04485G

22 Tiwari, D., Cardoso-Delgado, F., Alibhai, D., Mombrú, M. & Fermín, D. J. Photovoltaic Performance of Phase-Pure Orthorhombic BiSI Thin-Films. *ACS Appl. Energy Mater.* **2**, 3878–3885 (2019). https://doi.org/10.1021/acsaem.9b00544

23 Sasaki, Y. Photoconductivity of a Ferroelectric Photoconductor BiSI. *J. J. Appl. Phys.* **4**, 614 (1965). https://doi.org/10.1143/JJAP.4.614

24 Demartin, F., Gramaccioli, C. M. & Campostrini, I. Demicheleite-(I), BiSI, a new mineral from La Fossa Crater, Vulcano, Aeolian Islands, Italy. *Mineralogical Magazine* **74**, 141–145 (2010). https://doi.org/10.1180/minmag.2010.074.1.141

25 Ran, Z. *et al.* Bismuth and antimony-based oxyhalides and chalcohalides as potential optoelectronic materials. *npj Comp. Mater.* **4**, 14 (2018). https://doi.org/10.1038/s41524-018-0071-1

26 Liu, P. *et al.* Electronic structures and band alignments of monolayer metal trihalide semiconductors $MX_3$. *J. Mater. Chem. C* **5**, 9066–9071 (2017). https://doi.org/10.1039/C7TC03003G

27 Brandt, R. E. *et al.* Investigation of Bismuth Triiodide ($BiI_3$) for Photovoltaic Applications. *J. Phys. Chem. Lett.* **6**, 4297–4302 (2015). https://doi.org/10.1021/acs.jpclett.5b02022

28 Lintereur, A. T., Qiu, W., Nino, J. C. & Baciak, J. Characterization of bismuth tri-iodide single crystals for wide band-gap semiconductor radiation detectors. *Nuclear Instruments and Methods in Physics Research Section A: Accelerators, Spectrometers, Detectors and Associated Equipment* **652**, 166–169 (2011). https://doi.org/https://doi.org/10.1016/j.nima.2010.12.013

29 Du, M.-H. & Singh, D. J. Enhanced Born charges in III-VII, IV-$VII_2$, and V-$VII_3$ compounds. *Phys. Rev. B* **82**, 045203 (2010). https://doi.org/10.1103/PhysRevB.82.045203

30 Prabhakar, T. *et al.* Effects of growth process on the optical and electrical properties in Al-doped ZnO thin films. *J. Appl. Phys.* **115**, 083702 (2014). https://doi.org/10.1063/1.4866997

31 Otamiri, J. C., Andersson, S. L. T. & Andersson, A. Ammoxidation of toluene by $YBa_2Cu_3O_{6+x}$ and copper oxides: Activity and XPS studies. *Applied Catalysis* **65**, 159–174 (1990). https://doi.org/https://doi.org/10.1016/S0166-9834(00)81595-X

32 Velásquez, P. *et al.* A Chemical, Morphological, and Electrochemical (XPS, SEM/EDX, CV, and EIS) Analysis of Electrochemically Modified Electrode Surfaces of Natural Chalcopyrite ($CuFeS_2$) and Pyrite ($FeS_2$) in Alkaline

Solutions. *J. Phys. Chem. B* **109**, 4977–4988 (2005). https://doi.org/10.1021/jp048273u

33 Poulston, S., Parlett, P. M., Stone, P. & Bowker, M. Surface Oxidation and Reduction of CuO and $Cu_2O$ Studied Using XPS and XAES. *Surface and Interface Analysis* **24**, 811–820 (1996). https://doi.org/10.1002/(sici)1096-9918(199611)24:12

34 Wang, D., Miller, A. C. & Notis, M. R. XPS Study of the Oxidation Behavior of the $Cu_3Sn$ Intermetallic Compound at Low Temperatures. *Surface and Interface Analysis* **24**, 127–132 (1996). https://doi.org/10.1002/(sici)1096-9918(199602)24:2<127::Aid-sia110>3.0.Co;2-z

35 Biesinger, M. C. Advanced analysis of copper X-ray photoelectron spectra. *Surface and Interface Analysis* **49**, 13251334 (2017). https://doi.org/https://doi.org/10.1002/sia.6239

36 Romand, M., Roubin, M. & Deloume, J. P. ESCA studies of some copper and silver selenides. *Journal of Electron Spectroscopy and Related Phenomena* **13**, 229–242 (1978). https://doi.org/https://doi.org/10.1016/0368-2048(78)85029-4

37 Qiu, X., Ji, S., Chen, C., Liu, G. & Ye, C. Synthesis, characterization, and surface-enhanced Raman scattering of near infrared absorbing $Cu_3SbS_3$ nanocrystals. *CrystEngComm* **15**, 10431–10434 (2013). https://doi.org/10.1039/C3CE41861H

38 Hsiang, H.-I., Yang, C.-T. & Tu, J.-H. Characterization of $CuSbSe_2$ crystallites synthesized using a hot injection method. *RSC Adv.* **6**, 99297-99305 (2016). https://doi.org/10.1039/C6RA20692A

39 Wang, C. *et al.* Reactive close-spaced sublimation processed $CuSbSe_2$ thin films and their photovoltaic application. *APL Mater.* **6**, 084801 (2018). https://doi.org/10.1063/1.5028415

40 Delobel, R., Baussart, H., Leroy, J.-M., Grimblot, J. & Gengembre, L. X-ray photoelectron spectroscopy study of uranium and antimony mixed metal-oxide catalysts. *Journal of the Chemical Society, Faraday Transactions 1: Physical Chemistry in Condensed Phases* **79**, 879–891 (1983). https://doi.org/10.1039/F19837900879

41 Guo, X. *et al.* Air-stable bismuth sulfobromide (BiSBr) visible-light absorbers: optoelectronic properties and potential for energy harvesting. *J. Mater. Chem. A* **11**, 22775–22785 (2023). https://doi.org/10.1039/D3TA04491B

42 Motti, S. G. *et al.* Exciton Formation Dynamics and Band-Like Free Charge-Carrier Transport in 2D Metal Halide Perovskite Semiconductors. *Adv. Funct. Mater.* **33**, 2300363 (2023). https://doi.org/https://doi.org/10.1002/adfm.202300363

43 Sadhanala, A. *et al.* Preparation of Single-Phase Films of $CH_3NH_3Pb(I_{1-x}Br_x)_3$ with Sharp Optical Band Edges. *J. Phys. Chem. Lett.* **5**, 2501–2505 (2014). https://doi.org/10.1021/jz501332v

44 Buizza, L. R. V. *et al.* Charge-Carrier Mobility and Localization in Semiconducting $Cu_2AgBiI_6$ for Photovoltaic Applications. *ACS Energy Lett.* **6**, 1729–1739 (2021). https://doi.org/10.1021/acsenergylett.1c00458

45 Wright, A. D. *et al.* Ultrafast Excited-State Localization in $Cs_2AgBiBr_6$ Double Perovskite. *J. Phys. Chem. Lett.* **12**, 3352–3360 (2021). https://doi.org/10.1021/acs.jpclett.1c00653

46 Wehrenfennig, C., Eperon, G. E., Johnston, M. B., Snaith, H. J. & Herz, L. M. High Charge Carrier Mobilities and Lifetimes in Organolead Trihalide Perovskites. *Adv. Mater.* **26**, 1584–1589 (2014). https://doi.org/https://doi.org/10.1002/adma.201305172

47 Joyce, H. J., Boland, J. L., Davies, C. L., Baig, S. A. & Johnston, M. B. A review of the electrical properties of semiconductor nanowires: insights gained from terahertz conductivity spectroscopy. *Semiconductor Science and Technology* **31**, 103003 (2016). https://doi.org/10.1088/0268-1242/31/10/103003

48 Bardeen, J. & Shockley, W. Deformation Potentials and Mobilities in Non-Polar Crystals. *Phys. Rev.* **80**, 72–80 (1950). https://doi.org/10.1103/PhysRev.80.72

49 Wei, S. H. & Zunger, A. Predicted band-gap pressure coefficients of all diamond and zinc-blende semiconductors: Chemical trends. *Phys. Rev. B* **60**, 5404–5411 (1999). https://doi.org/10.1103/PhysRevB.60.5404

50 Klarbring, J., Hellman, O., Abrikosov, I. A. & Simak, S. I. Anharmonicity and Ultralow Thermal Conductivity in Lead-Free Halide Double Perovskites. *Phys. Rev. Lett.* **125**, 045701 (2020). https://doi.org/10.1103/PhysRevLett.125.045701

51 Yin, G.-X., Lv, S.-J. & Wang, H.-Y. First principles study of the electronic, elastic and vibrational properties of BiOI. *Solid State Communications* **336**, 114419 (2021). https://doi.org/https://doi.org/10.1016/j.ssc.2021.114419

52 Liu, Y., Eddie Chua, K. T., Sum, T. C. & Gan, C. K. First-principles study of the lattice dynamics of $Sb_2S_3$. *Phys. Chem. Chem. Phys.* **16**, 345–350 (2014). https://doi.org/10.1039/C3CP53879F

53 Lehner, A. J. *et al.* Crystal and Electronic Structures of Complex Bismuth Iodides $A_3Bi_2I_9$ (A = K, Rb, Cs) Related to Perovskite: Aiding the Rational Design of Photovoltaics. *Chem. Mater.* **27**, 7137–7148 (2015). https://doi.org/10.1021/acs.chemmater.5b03147

54 Shi, H., Ming, W. & Du, M.-H. Bismuth chalcohalides and oxyhalides as optoelectronic materials. *Phys. Rev. B* **93**, 104108 (2016).

https://doi.org/10.1103/PhysRevB.93.104108
55 Du, M. H. Efficient carrier transport in halide perovskites: theoretical perspectives. *J. Mater. Chem. A* **2**, 9091–9098 (2014). https://doi.org/10.1039/C4TA01198H
56 Gaillac, R., Pullumbi, P. & Coudert, F.-X. ELATE: an open-source online application for analysis and visualization of elastic tensors. *Journal of Physics: Condensed Matter* **28**, 275201 (2016). https://doi.org/10.1088/0953-8984/28/27/275201
