# Supplementary material for: Structural and electronic features enabling delocalized charge-carriers in CuSbSe2
Source: Nat Commun. 2025 Jan 2;16:65. doi: 10.1038/s41467-024-55254-2 (PMC11697385; doi:10.1038/s41467-024-55254-2)
Supplement: Supplementary file 2 — Description of Additional Supplementary Files [file 41467_2024_55254_MOESM2_ESM.pdf]

Description of Additional Supplementary Files:

Supplementary Data 1: Optimized structure of $CuSbSe_2$ from calculations with PBE functional

Supplementary Data 2: Optimized structure of $CuSbSe_2$ from calculation with r2SCAN functional

Supplementary Data 3: Optimized structure of $CuSbSe_2$ from calculations with HSE06 functional
